# Therapeutic Strategies and Biomarkers to Modulate PARP Activity for Targeted Cancer Therapy

**DOI:** 10.3390/cancers12040972

**Published:** 2020-04-14

**Authors:** Naveen Singh, S. Louise Pay, Snehal B. Bhandare, Udhaya Arimpur, Edward A. Motea

**Affiliations:** 1 School of Medicine, Department of Biochemistry and Molecular Biology, Indiana University, Indianapolis, IN 46202, USA; navsing@iu.edu (N.S.); slpay@iupui.edu (S.L.P); snebhan@iu.edu (S.B.B.); uarimpur@iu.edu (U.A.); 2 Simon Comprehensive Cancer Center, Indiana University, Indianapolis, IN 46202, USA

**Keywords:** PARP Inhibitors, beta-lapachone, NQO1, PARG, NAMPT, cancer therapeutics, DNA repair, cMET

## Abstract

Poly-(ADP-ribose) polymerase 1 (PARP1) is commonly known for its vital role in DNA damage response and repair. However, its enzymatic activity has been linked to a plethora of physiological and pathophysiological transactions ranging from cellular proliferation, survival and death. For instance, malignancies with *BRCA1/2* mutations heavily rely on PARP activity for survival. Thus, the use of PARP inhibitors is a well-established intervention in these types of tumors. However, recent studies indicate that the therapeutic potential of attenuating PARP1 activity in recalcitrant tumors, especially where PARP1 is aberrantly overexpressed and hyperactivated, may extend its therapeutic utility in wider cancer types beyond BRCA-deficiency. Here, we discuss treatment strategies to expand the tumor-selective therapeutic application of PARP inhibitors and novel approaches with predictive biomarkers to perturb NAD^+^ levels and hyperPARylation that inactivate PARP in recalcitrant tumors. We also provide an overview of genetic alterations that transform non-BRCA mutant cancers to a state of “BRCAness” as potential biomarkers for synthetic lethality with PARP inhibitors. Finally, we discuss a paradigm shift for the use of novel PARP inhibitors outside of cancer treatment, where it has the potential to rescue normal cells from severe oxidative damage during ischemia-reperfusion injury induced by surgery and radiotherapy.

## 1. Introduction

**Figure 1:**
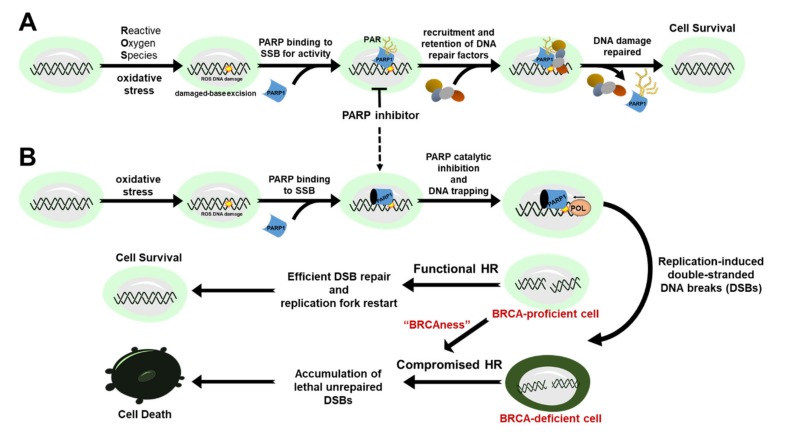
The role of PARP1 in DNA damage response and repair and cancer therapy. (**A**) PARP1 binds to single-strand breaks (SSB) for activity to target, recruit and retain critical DNA repair proteins at the sites of DNA lesions. (**B**) PARP inhibitors convert SSBs to lethal double-stranded breaks (DSBs) that are left unrepaired in BRCA-deficient cells due to a compromised homologous recombination (HR) repair consequently leading to cell death.

Maintenance of genomic integrity is vital to achieve normal cellular function and to prevent the development of diseases such as cancer [1]. At the heart of this intricate biological process are DNA repair factors that work harmoniously to scan, detect, and repair potentially deleterious damage to cellular genetic information. Indeed, the disruption of one or more DNA repair pathways compromises genetic stability and is a known mechanism of cancer initiation, development, and progression. Poly-(ADP-ribose) polymerase 1 (PARP1) is the classical and founding member of at least 17 human PARP enzymes that share the ability to catalyze the transfer of ADP-ribose units to target proteins to modulate chromatin structure, transcription, replication, DNA damage response and repair [2]. PARP1 is an abundant nuclear protein that acts as a DNA damage sensor and a facilitator of DNA repair pathway choice in response to cellular stress [3,4,5,6]. Specifically, it is involved in the repair of single-stranded DNA breaks (SSB) via the base-excision repair (BER) pathway. In BER, PARP1 functions to recruit other repair factors by binding to single-stranded DNA break intermediates. Additionally, it catalyzes the synthesis of poly(ADP)-ribose (PAR) chains to the acceptor proteins (e.g., histones and XRCC1), including itself, using nicotinamide adenine dinucleotide (NAD^+^) as the substrate and source of energy. The PARylated proteins then recruit and retain critical processing factors to the site of the lesion to facilitate the efficient repair of the SSB (Figure 1A) [7]. 

In the absence of PARP1, unrepaired SSBs are converted to double-stranded breaks (DSBs) during replication or the S-phase of the cell cycle [8]. Double-stranded DNA breaks are one of the most lethal forms of DNA damage induced by exogenous DNA damaging agents (e.g., ionizing radiation (IR) and chemotherapeutic agents) or endogenous replicative stress in fast proliferating cancer cells. Thus, accurate repair of DSBs is paramount to the growth and survival of all cells. Indeed, cells have evolved a range of DSB repair mechanisms. The two major repair pathways that have been studied extensively are homologous recombination (HR) and non-homologous end-joining (NHEJ) [9]. HR is largely error-free and more prevalently activated during and after DNA duplication when an identical chromatid is accessible as a template for repair. In contrast, NHEJ is active throughout the cell cycle and promotes direct ligation of DSB ends at the cost of small insertions, deletions, substitutions at the break, and even translocations that arise if DSBs from different parts of the genome are combined [10]. Cells utilize two mechanistically distinct end-joining pathways to process DNA DSBs [10,11,12]: Classical-NHEJ (c-NHEJ) leads to a minimal sequence alteration at the repair junctions, whereas alternative-NHEJ (alt-NHEJ, also known as microhomology-mediated end-joining (MMEJ) or back-up NHEJ) causes extensive genetic changes (deletions and insertions) that scar the break sites following ligation of DSB ends. 

Several studies have shown that loss-of-function mutation of canonical HR factors – such as breast cancer type 1 and 2 (BRCA1/2) susceptibility proteins that are commonly associated with breast and ovarian cancer [13,14,15] – promotes PARP1 hyperactivation in fast replicating cancer cells [16,17]. This suggests that hyperactivation of PARP1 is essential to facilitate the repair of potentially lethal DNA breaks for the survival of HR-defective (HRD) cancers. Indeed, *BRCA1/2* mutant cancers are selectively killed by PARP inhibitors (Figure 1B), which has led to the approval of these agents to treat HR-deficient ovarian and breast cancers. However, several studies have reported that certain cancers without a BRCA deficiency have significant clinical benefits to PARP1 inhibitors positing that PARP inhibitors could be expanded to a target population beyond BRCA-deficiency (e.g., *gBRCA* mutation carriers) [18,19,20,21]. 

This review article highlights treatment strategies to selectively target BRCA-proficient cancers by modulating PARP activity that alters PARP binding, PAR and NAD^+^ levels to induce tumor-selective cell death using predictive biomarkers for therapeutic response. We summarize how aberrant alterations of PARG, NAMPT, NQO1, cMET and “BRCAness” genes that have been shown to affect PARP activity in cancers could serve as prognostic biomarkers for targeted therapy. Finally, we briefly discuss innovative approaches for the use of novel PARP inhibitors to rescue injured normal cells from severe oxidative damage during ischemia-reperfusion injury that could be induced by surgery and radiotherapy.

## 2. Mechanism of Action for PARP Inhibitors

PARP inhibitors are designed to mimic the substrate-protein interactions of NAD^+^ within the ADP-ribose transferase (ART) catalytic core of PARP1-3, which are key DNA damage response sensors and transducers. The inhibitors compete with NAD^+^ binding site of PARP to inhibit PAR polymerization, which then hinders the recruitment and regulation of DNA repair factors and the eventual release of PARP from DNA damage. Two mechanisms are proposed to induce the lethality of PARP inhibitors: PARP catalytic inhibition and PARP trapping [22,23]. However, the relative contributions of these two pathways in mediating the lethality of PARP inhibitors remain enigmatic. For example, there is evidence suggesting that the differences in the trapping potential of PARP1 to the DNA are more efficient at killing HR-deficient cells [22,23]. While there has been no evidence that this mechanism exists with clinically-used PARP inhibitors, a recent study has demonstrated that certain non-hydrolysable NAD^+^ analogs (e.g., benzamide adenine dinucleotide, BAD) binding at the catalytic site of PARP1 could greatly enhance the binding affinity of PARP1 at sites of DNA damage that prevents its release [24]. Mechanistically, this analog competes with NAD^+^ at the catalytic binding site that then stabilizes a conformation that induces the DNA binding domain of PARP1 to be locked on a DNA break with a significantly better binding affinity (~10-fold) – a phenomenon known as “reverse allostery.” While most PARP inhibitors (e.g., Rucaparib) bind at the catalytic site, not all of them are “created equal.” In fact, the relative contribution of DNA trapping induced by PARP inhibitors contributes significantly to the toxicity induced by these agents in cancers that are deficient in HR pathways [23,25,26,27]. Some of them have variable inhibitory effects on the PARP isoforms other than PARP1, and some may have more off-target effects on kinases than others. Regardless, PARP inhibitors are now being explored for use in several cancers, even in cancers beyond *BRCA* mutations [25,28,29,30]. Interestingly, a recent study has reported a correlation between therapeutic response to PARP inhibition and the patterns of ADP-ribosylation (i.e., amount of PARylation) in a panel of ovarian cancers, suggesting that ADP-ribosylation may be a useful biomarker for HR deficiency and sensitivity to PARP inhibitors regardless of BRCA status [31]. While most of the PARP inhibitors have been suggested to specifically inhibit PARP1 and/or PARP2, our limited understanding of the overlapping and non-overlapping functions and cross-talks among all of the PARP family members still raises the problem of target specificity and the risk of unintended effects and consequences promoted by the targeting of other PARP family members. 

## 3. Approved PARP Inhibitors for Targeted Cancer Therapy

PARP inhibitors such as Olaparib [32,33], Rucaparib [34], and Niraparib [35] have been approved by the Food and Drug Administration (FDA) and the European Medicine Agency (EMA) for the treatment of adults with deleterious or suspected deleterious germline *BRCA*-mutated ovarian cancer in different settings [36]. Moreover, these agents have also been approved for maintenance therapy in platinum-sensitive high-grade ovarian cancer (HGOC), which has a high recurrence rate and an extremely poor prognosis following relapse [37]. Currently, PARP inhibitors are being evaluated in clinical trials in other cancer settings such as nonsmall cell lung cancer, pancreatic cancer, and gastric cancer (ClinicalTrials.gov Identifiers NCT01082549, NCT02184195, NCT03427814, respectively).

Olaparib (LYNPARZA, AstraZeneca and Merck) was the first PARP inhibitor to receive FDA approval for indications in breast cancer and pancreatic cancer, and has since been approved for metastatic breast cancer in patients with germline *BRCA1* or *BRCA2* (*gBRCA1/2*) mutations. In 2009, Olaparib was deemed safer than conventional chemotherapeutics, with mild and reversible side effects during clinical trials [26]. It was subsequently determined to have anti-tumor activity in advanced ovarian cancer patients with *gBRCA1/2* mutations, particularly with primary tumors that are sensitive to platinum [18,32,38,39,40]. The phase III OlympiAD trial found a 60% reduction in metastatic *BRCA1/2* mutant breast tumor size with Olaparib in comparison to 29% in patients receiving conventional chemotherapy. The FDA approved Olaparib in ovarian and breast patients with *gBRCA* mutations in 2014 and 2018, respectively [27,41,42,43]. 

Rucaparib (RUBRACA, Clovis Oncology, Inc.) was granted accelerated approval by the FDA in 2016 for the treatment of adult ovarian cancer patients with germline and/or somatic *BRCA*-mutations previously treated with two or more prior lines of chemotherapy. This agent is also approved as a maintenance monotherapy for adult patients with recurrent ovarian cancer who have established complete or partial response (CR/PR) to platinum-based chemotherapy. In 2018, Rucaparib gained FDA approval for the maintenance treatment of advanced or recurrent ovarian, fallopian tube, and primary peritoneal cancer in patients who have received platinum-based chemotherapies, as well as in patients with germline or somatic *BRCA1/2* mutations following two or more treatments with conventional chemotherapy [44,45]. 

Niraparib (ZEJULA, Tesaro, Inc.) was approved by the FDA and EMA in 2017. Compared to Olaparib, this agent results in longer progression-free survival in patients with *BRCA1/2*-mutant tumors [35]. Niraparib also increases progression-free survival in patients with wild-type *BRCA1/2* ovarian cancers, though less effectively than in patients with *BRCA* mutations [35]. Niraparib, however, is associated with more severe side effects than other PARP inhibitors, with patients experiencing thrombocytopenia (33.8%), anemia (25.3%), and neutropenia (19.6%) [35]. In 2019, the FDA approved Niraparib for homologous recombination-deficient (HRD) patients with advanced ovarian, fallopian tube, or primary peritoneal cancer previously treated with three or more chemotherapies [46].

Talazoparib (TALZENNA, Pfizer, Inc.) is a new active PARP inhibitor approved by the FDA in 2018 for patients with deleterious or suspected deleterious *gBRCA*-mutated, HER2-negative local advanced or metastatic breast cancer [47]. Compared to other PARP inhibitors, Talazoparib is still at an early stage of clinical development in gathering evidence to support its use on the treatment of epithelial ovarian cancer.

## 4. Harnessing the Power of PAR and PARG Inhibition for Cancer Therapy

Poly(ADP)-ribosylation (PARylation) is a covalent and reversible posttranslational modification (PTM) of acceptor proteins catalyzed by PARPs, particularly in response to DNA damage and oxidative stress [48,49]. PARylation is also a critical signaling PTM for other biological transactions such as transcription, cell cycle regulation, and genome maintenance [50,51,52]. Intracellular PAR levels are very low in unstressed cells due to low enzymatic activity of PARP. When PARP1 is activated following DNA damage, PAR formation increases and consequently depletes intracellular NAD^+^ and ATP levels [53]. Accumulation of PAR and loss of NAD^+^ and ATP can lead to severe metabolic dysfunction and eventual cell death. Thus, the balance between ADP-ribose synthesis (i.e., PAR writers) and degradation (i.e., PAR erasers) is critical for the coordination of various cellular response pathways for survival [54]. 

**Figure 2:**
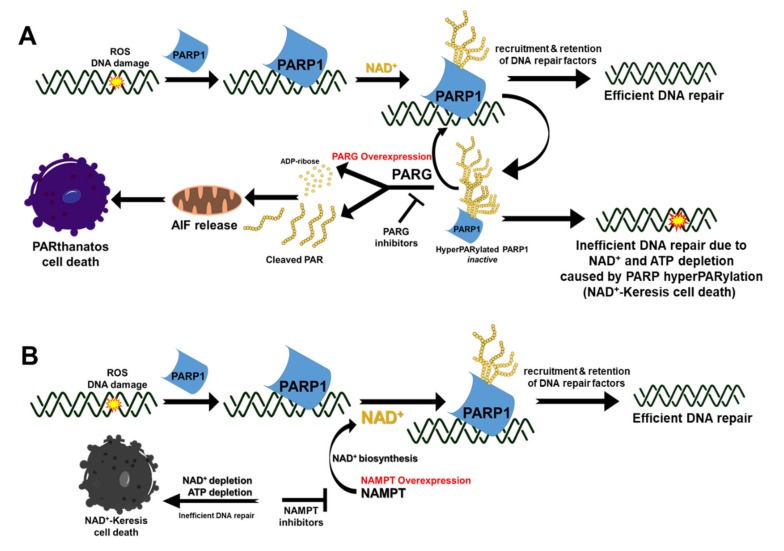
Pharmacological modulation of PAR ad NAD^+^ in cancers for therapy. (**A**) Mechanism of PARthanatos mediated by the translocation of cleaved PAR from the nucleus to the cytosol and mitochondria to induce the release of AIF that translocates into the nucleus to initiate death in PARG-overexpressing cancers. Alternatively, breakdown and recycling of PAR can be prevented by inhibition of PARG to enhance NAD^+^ depletion caused by PARP hyperactivation, ultimately starving the cell of ATP needed for various critical cellular processes (e.g., DNA repair). (**B**) In cancer cells that overexpress NAMPT, the use of NAMPT inhibitors interfere with generation of intracellular NAD^+^ levels that compromise PARP activity, consequently resulting in impaired repair of DNA damage and NAD^+^-Keresis.

Poly(ADP-ribose) glycohydrolase (PARG) is an endo- and exo-glycohydrolase that rapidly catalyzes the degradation of PAR polymerized by PARP1 to coordinate DNA repair [48,49]. It is comprised of an N-terminal regulatory domain required for recruitment to DNA damage sites [55], a C-terminal catalytic domain, and a central mitochondrial-targeting sequence [56]. PARG hydrolyzes the glycosidic bonds between ADP-ribose units producing free ADP-ribose and PAR oligomers (Figure 2A) [57], which play distinct biological roles in cellular processes. Free ADP-ribose is predominantly involved in energy catabolism, calcium signaling, and protein glycation [57]. The long-chain of free PAR formed by PARG cleavage has been shown to interact with the mitochondrial proteins triggering a unique intrinsic PAR-mediated cell death program known as PARthanatos (Figure 2A**) [58,59]**. Mechanistically, a cell commits to PARthanatos when free PAR (> 60 ADP-ribose units) migrates from the nucleus to the cytosol, which then triggers the translocation of PAR-bound apoptosis-inducing factor (AIF) from the mitochondria to the nucleus to activate the cell death process (Figure 2A) [60]. Early events in PAR-mediated cell death include loss of mitochondrial membrane potential and mitochondrial permeability transition [61]. While caspase activation has been demonstrated to act as a bystander in PAR-dependent cell death, this caspase-independent process shares cytological and morphological characteristics of both necrosis and apoptosis [58,59].

ADP-ribose hydrolases (ARH) can catalyze PAR hydrolysis but to a lesser extent than PARG, which catalyzes the majority (~90%) of PAR catabolism [62]. PARG is aberrantly overexpressed in most human cancers, suggesting that PARG is required for tumorigenesis and cancer survival [63]. Therefore, inducing cell death via PARG inhibition and manipulation of the PAR cycle through PARP1/PARG inhibition is an attractive target for cancer therapy (Figure 2). Available PARG inhibitors consist of DNA intercalators, tannins, and substrate analogs. DNA intercalators (e.g., Tilorene, GPI116552, GPI18214, Ethacridine) indirectly inhibit PARG by forming complexes with its substrate, PAR [64,65,66]. Hydrolyzable tannins (e.g., Nobotanin B, Oenothenin B, gallotannin) inhibit PARG in vitro by competing with the PAR substrate [67,68,69]; however, poor membrane permeability, poor bioavailability, and toxicity of these compounds hinder their usage. Adenosinediphosphate (hydroxymethyl) pyrrolidinediol (ADP-HPD), a nonhydrolyzable-analogue of ADP-ribose, has been shown to be a potent competitive PARG inhibitor [66]. Other reversible PARG inhibitors under development include modified salicylanilide pharmacophore [70] and rhodanine-based small molecule analogs (RBPIs) [70]. Recently, Pillay et al. [71] demonstrated that PARG inhibitor, PDD00017273, mimics poly(ADP-ribose) polymerase (PARP) inhibitor therapy in ovarian cancer cells by exacerbating the formation of hyperPARylated-PARP1 that severely compromises PARP activity. Mechanistically, PARG inhibitors prevent the recycling of hyperPARylated-PARP1 to its more active form for efficient DNA damage response and repair. In support of this, survival studies of certain human cancer cell lines with *PARG* knockdown synergistically enhances the lethality to DNA-damaging agents such as alkylating agents [72,73] and cisplatin [72]. Overall, pharmacological targeting of PARG is a promising therapeutic target and future investigations are required to develop effective strategies to manipulate the dynamic PAR formation and break-down process in response to DNA damage. The relative low abundance of PARG in normal cells, compared to tumors with aberrant PARG overexpression, makes it an attractive target in cancer therapeutics for tumor-selective drug response. Moreover, unlike the human PARP family with 17 related enzymes with almost similar catalytic subunit – PARG is a unique mammalian protein without any paralogs, which may offer fewer off-target effects than PARP inhibitors.

## 5. Targeting NAD^+^ for Cancer Therapy

NAD^+^ is a co-enzyme that mediates redox reactions by acting as an electron carrier and a substrate in metabolic pathways including glycolysis, the tricarboxylic acid cycle (TCA), oxidative phosphorylation, and serine biosynthesis [74]. It is a cofactor for PARP and plays a key role in energy production, cell signaling, redox homeostasis, DNA repair, gene expression, and the stress response. Many of these processes are disrupted in cancer, which alters NAD^+^ production and consumption [75,76]. Nicotinamide phosphoribosyltransferase (NAMPT) and nicotinamide mononucleotide adenylyltransferase (NMNAT) enzymes regulate the salvage pathway critical for controlling intracellular NAD^+^ levels [77]. Increased NAD^+^ and aberrant NAMPT overexpression promotes glycolysis, which fuels the growth and survival of cancer cells [78,79]. Indeed, NAMPT inhibitors suppress cancer cell proliferation by inhibiting glycolysis [79,80,81,82,83]. 

Overexpression of NAMPT is observed in several types of malignant tumors, which supplies back-up NAD^+^ to sustain cellular proliferation and promote resistance to therapeutic agents [84,85,86,87,88,89]. NAMPT as a predictive biomarker for tumor-selective targeting of NAD^+^ metabolism is, therefore, a promising therapeutic strategy (Figure 2B). Mechanistically, NAMPT inhibitors induce cell death in cancer cells by depleting intracellular NAD^+^ and ATP levels and inhibiting glycolysis and glucose uptake [90,91]. Examples of NAMPT inhibitors include FK866, GMX-1777/8, STF-31, STF-118804, GNE-617, GNE-618, LSN3154567, KPT-9274. Vacor adenine dinucleotide (VAD) is an NAD^+^ analog that has been shown to inhibit both NAMPT and NMNAT, leading to necrotic cell death through NAD^+^ depletion (NAD^+^-Keresis, Figure 2B), glycolytic block, and energy failure [82,92,93,94,95,96,97,98]. Several NAMPT inhibitors have been investigated in the clinic as a monotherapy such as FK866 and GMX-1777/8. However, thrombocytopenia was demonstrated to be the dose-limiting toxicity in these clinical trials [99,100]. Though NAMPT inhibitors as a monotherapy are promising candidates for cancer therapy, drug resistance and toxicity to normal tissues when used at high concentration remain a major hurdle [99,101,102,103]. Perhaps, a combination approach with tumor-selective DNA damaging agents (e.g., NQO1-bioactivatable agents, vide infra in Section 6) may overcome this limitation to improve the tumor-selectivity of NAMPT inhibitors by severely depleting the NAD^+^ needed by PARPs for efficient DNA repair and survival [82,104]. 

## 6. Leveraging NQO1 as a Biomarker for Tumor-Selective Use of PARP Inhibitors with NQO1-Bioactivatable Drugs 

**Figure 3:**
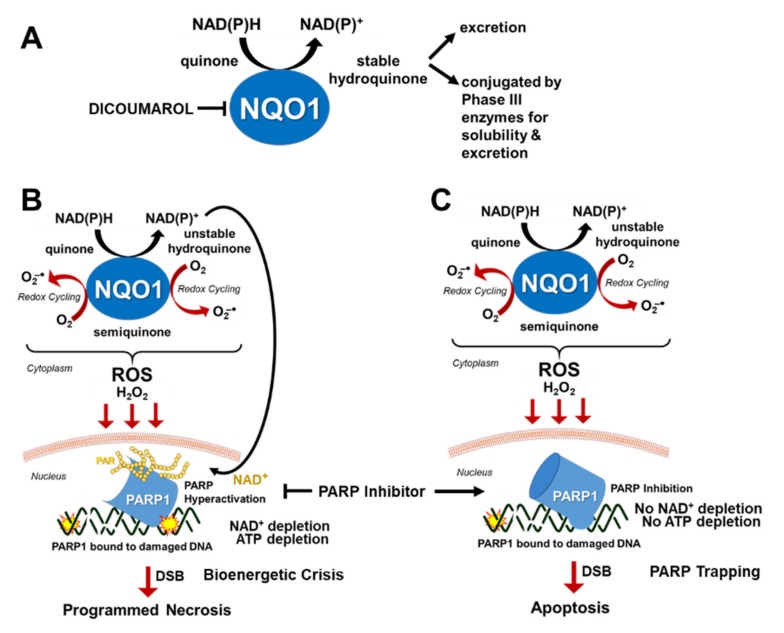
Strategy for tumor-selective use of PARP inhibitors in solid tumors. (**A**) The role of NQO1 in detoxifying certain quinones for cellular efflux. (**B**) The role of NQO1 in bioactivating certain quinones to induce toxicity in NQO1(+) cancer cells. Note that the stability of hydroquinones determine whether bioreduction by NQO1 leads to detoxification or toxicity. (**C**) Mechanism of tumor-selective synergistic cell death induced by combination of PARP inhibitor and NQO1-bioactivatable agents in NQO1(+) cells.

NAD(P)H:Quinone Oxidoreductase 1 (NQO1, DT-diaphorase) is a cytoplasmic Phase II detoxification enzyme that utilizes NAD(P)H to reduce certain quinones to stable hydroquinones via two-electron reductions that are readily conjugated by Phase III transporters for cellular efflux (Figure 3A) [105]. NQO1 is abnormally overexpressed in most malignant tumors, which contributes to drug resistance by metabolizing xenobiotics to their inactive forms or chemical structures that are excreted out of the cell for chemoprotection. Indeed, the addition of an NQO1-inhibitor (e.g., dicoumarol) in cancers could sensitize the anti-cancer effects of certain agents by inhibiting resistance due to drug efflux. 

Certain quinones are bioactivated by NQO1 to create a rapid increase in reactive oxygen species (ROS), particularly hydrogen peroxide (H_2_O_2_), which can permeate through the nucleus to induce the formation of toxic DNA damage leading to cell death (Figure 3B) [106,107,108,109,110]. For example, β-lapachone (β-lap) is a soluble ortho-naphthoquinone with potent anti-tumor and radiosensitizing activity only in the presence of high NQO1 activity [108,109,110,111,112,113,114], which is only noted in most malignant tumors but not in normal tissues [109]. Mechanistically, the two-electron reductase capacity of NQO1 catalyzes the oxidoreduction of β-lap to an inherently unstable hydroquinone, which automatically and rapidly gets reverted back to its original quinone form as it undergoes a two-step oxidation (Figure 3B) [108]. This produces what is termed as a “futile cycle” of oxidation and produces a significant level of reactive oxygen species such as superoxide radicals (O_2_^−^) that are rapidly catalyzed by superoxide dismutases to hydrogen peroxide in the cytoplasm (Figure 3B) [115]. This stable form of ROS can easily penetrate through the nucleus and is converted to genotoxic hydroxyl radicals via the Fenton reaction process, which causes massive formation of oxidative DNA base damage and SSBs (Figure 3B) [109,116]. The surge of this specific types of DNA damage in the nucleus causes hyperactivation of PARP1, which is an NAD^+^-dependent enzyme, to a level that consequently leads to NAD^+^-Keresis death due to the depletion of critical biological energy sources, NAD^+^ and ATP [82,107,109]. Another possibility is that PARP hyperactivation could lead to an NAD^+^-independent glycolytic and bioenergetics crisis that is presumably caused by PAR-dependent inhibition of glycolysis that occurs through the inhibition of hexokinase leading to a specific form of cell death termed “PARthanathos” [117]. Future studies are required to firmly establish the contribution of PARP hyperactivation, NAD^+^, or free PAR in specific types of cell death. Regardless, the use of NQO1-bioactivatable agents to modulate PARP activity, NAD^+^ and PAR to induce cell death is a viable strategy to treat cancer. As NQO1 is typically overexpressed in tumors but not at a significant level in normal healthy cells [109], the application of NQO1-bioactivatable agents is an attractive possibility for tumor-selective generation of H_2_O_2_-induced DNA damages that hyperactivate PARP and deplete NAD^+^ to cause eventual cell death, which is vital for developing safe and effective drugs with minimal off-target effects. 

While β-lap (ARQ761 in clinical form) shows potential as a single-agent therapeutic [107,118], it has also been associated with hemolytic anemia at a dose of 450 mg/m^2^ [119]. Therefore, increasing its efficacy at a lower dose is therapeutically advantageous. Due to the role of PARP hyperactivation in the mechanism of action of β-lap, PARP inhibitors have been a central focus in improving the efficacy and reducing the toxicity of β-lap for use in NQO1(+) cancers. PARP inhibition followed by β-lap treatment alters the mechanism of β-lap function by blocking the repair of damaged DNA and inhibiting PARP1 hyperactivation, slowing down the process of NAD^+^ depletion as a result, and extending the cycling of β-lap by NQO1 to maximize hydrogen peroxide production and amplifying DNA damage in a tumor-selective manner (Figure 3C) [109]. Instead of dying by caspase-independent NAD^+^-Keresis, cell death from exposure to β-lap following PARP1 inhibition occurs through caspase-dependent apoptosis, presumably due to the availability of NAD^+^ and ATP (Figure 3C) [109]. As NQO1 will continue cycling β-lap until the drug is depleted or the required source of NAD(P)H is depleted, blocking the DNA repair process which leads to NAD^+^ depletion following β-lap treatment prolongs the efficacy of the drug, allowing for lower doses to be used with the synergistic effect, minimizing the potential for hemolytic anemia. 

The use of NQO1-bioactivatable drug (e.g., β-lap) with PARP inhibitors have been shown to significantly expand the clinical application of PARP inhibitors outside of *BRCA1/2* mutant cancers for which the majority of PARP inhibitors are currently approved. The prevalence of NQO1 overexpression in such a wide variety of malignant cancers (>90% in pancreatic cancers with KRAS mutation, >80% nonsmall cell lung cancer (NSCLC), >60% breast cancer, >60% prostate cancer, >45% head and neck, and >60% colon cancers [109]) suggests that this combination therapy may have significant future potential, especially in cancers in which prognosis is currently poor, such as head and neck cancer, pancreatic cancer and NSCLC [117,120,121,122,123,124,125,126].

## 7. Targeting cMET to Attenuate PARP1 Activity

Many studies have shown that most malignant tumors have increased levels of reactive oxygen species (ROS), particularly hydrogen peroxide (H_2_O_2_), compared to their normal counterparts. These genotoxic reactive species cause oxidative DNA damage and single-stranded DNA breaks that stimulate PARP activity in DNA repair [127]. Interestingly, Du and colleagues [128] have shown that cMET, which is a receptor tyrosine kinase, can further stimulate the activity of PARP1 in cancers to survive the lethal effects of ROS-induced DNA damage. In cancer, cMET is overexpressed and its abnormal activation can promote the development and progression of multiple cancers [129].

**Figure 4:**
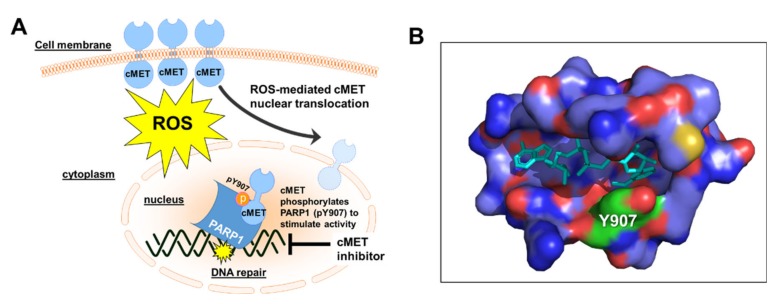
Attenuation of PARP Activity by cMET inhibition. (**A**) cMET enhances PARP activity during oxidative stress. Inhibition of cMET is an excellent strategy for inhibiting PARP1-mediated DNA repair and synergy with PARP inhibitors. (**B**) A snapshot of non-hydrolyzable NAD^+^ analog (in cyan) bound to residues in PARP1 catalytic active site within 6Å from the ligand (PDB ID: 6 bhv, processed via PyMOL Molecular Graphics System). Phosphorylation of PARP1 residue Y907 (shown in green) inhibits binding of PARP inhibitors.

Mechanistically, the high level of ROS in cancer promotes the activation and translocation of cMET into the nucleus where it directly binds and phosphorylates the tyrosine residue 907 (Y907) of PARP1 to enhance its enzymatic activity (Figure 4A) [128,130,131]. Indeed, Du and colleagues have demonstrated that phosphorylated PARP1 (pY907) showed a higher level of PAR production than non-phosphorylated PARP1 in vitro, thereby highlighting the critical role of cMET in enhancing PARP1 activity in DNA repair [128,130,131]. Moreover, the phosphorylation of this specific residue (pY907) has been suggested to significantly decrease the binding of clinically-relevant PARP inhibitors due to a potential steric hindrance (Figure 4B) [128]. Indeed, c-MET-mediated phosphorylation of PARP1 at Y907 leads to PARP inhibitor resistance, which could be potentiated when combined with cMET inhibitors (e.g., crizotinib and foretinib) [128]. Thus, inhibition of cMET is an attractive strategy to indirectly block PARP1-mediated DNA repair and enhance the therapeutic effects of PARP inhibitors in cancer cells [128]. However, the mechanistic basis for requiring Y907 phosphorylation to promote H_2_O_2_-induced PARP1 activity remains to be firmly established. A recent report also suggests that phosphorylation of PARP1 by EGFR and cMET heterodimer contributes to PARP1 inhibitor resistance. Hence, combination treatment consisting of EGFR, cMET, and PARP1 inhibitors posits a novel therapeutic strategy in cancer treatment involving overexpression of PARP1/EGFR/cMET, which are frequent alterations in most solid tumors [132]. 

## 8. Targeting of PARP Activity in Non-Oncological Events

PARP inhibition or gene deletion has been shown to attenuate tissue injury associated with ischemia-reperfusion injury and inflammation that could arise during tumor surgical resection and radiation therapy [133,134]. During this non-oncological event, the catalytic activity of PARP1 becomes hyperactivated and consequently leads to NAD^+^ depletion. This process forces NAD^+^ replenishment through the salvage pathway, which decreases cellular ATP levels and results in bioenergetic crisis, eventually leading to necrotic cell death that is typically associated with inflammation [3,135]. Thus, inhibition of PARP activity could have protective effects by dramatically reducing NAD^+^ consumption and preventing energetic failure and the consequent necrotic cell death. While the trapping mechanism induced by specific PARP inhibitors might be advantageous for the treatment of certain cancers, this mechanism of action might not be optimal to rescue normal cells from severe oxidative damage during ischemia-reperfusion injury (e.g., ischemia of the lung due to ionizing radiation [136] and cerebral ischemia during surgery [137]).

**Figure 5:**
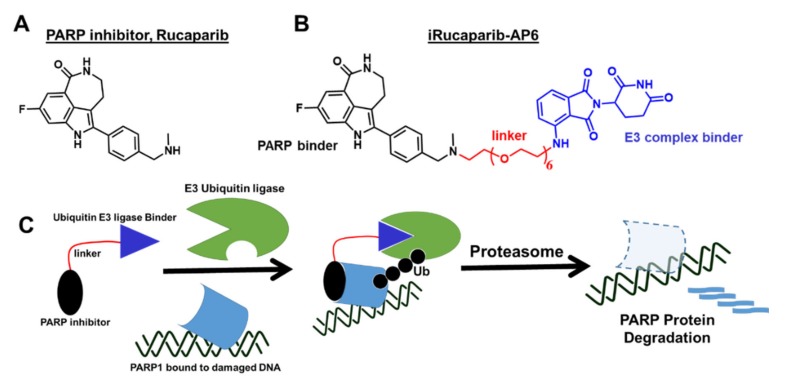
Development of a novel “PARP degrader” molecule as a biochemical probe and therapeutic agent. (**A**) Chemical structure of Rucaparib. (**B**) Chemical structure and elements of iRucaparib-AP6. (**C**) Simplified representation of the mechanism of action for a novel proteolysis targeting chimeric (PROTAC) molecule, iRucaparib-AP6.

For non-oncological indications, the safety profile of most FDA-approved PARP inhibitors would be expected to be better if it had less PARP trapping activity to restore the viability of normal tissue damage from injury caused by ischemia-reperfusion. To circumvent the cytotoxic effects of PARP trapping, the Yu group [138] developed a strategy to decrease PARP activity using a novel lead compound, iRucaparib-AP6 (Figure 5), which promotes PARP degradation upon binding that mimics PARP1 genetic deletion. Mechanistically, the Rucaparib component of the small molecule specifically binds PARP at the site of damaged DNA, but it is attached to a linker with a ligand that brings the E3 ligase in close proximity to ubiquitinate PARP and subsequently gets degraded through the proteasome pathway (Figure 5C). Unlike PARP trapping, this strategy of modulating PARP activity protects cells against genotoxic stress-induced cell death [138] that could have potential clinical applications for the treatment of ischemia-reperfusion injury and neurodegeneration. However, further development is needed to firmly establish the mechanism of action and evaluate its protective effects to treat the aforementioned non-oncological indications. 

To summarize, the enzymatic function of PARP1 – which is to catalyze the PARylation of a large number of PAR-acceptor proteins (including itself) – becomes hyperactivated upon detection of genotoxic stresses associated with various pathological conditions such as cancer, ischemia-reperfusion injury (e.g., myocardial infarction), inflammatory diseases (e.g., colitis, arthritis, asthma), vascular diseases (e.g., diabetic complications, atherosclerosis), and neurodegeneration. Thus, modulation of PARP activity using a novel class of PARP activity inhibitors (e.g., iRucaparib-AP6) with little to no “trapping” potential may be a promising strategy to ameliorate certain pathological conditions that are aimed to heal instead of killing the severely injured normal tissues (e.g., inflamed tissues). Indeed several studies have looked at the protective effects of PARP inhibitors in lung transplantation [139], neurodegeneration [140], renal injury [141], aging [142], and acute pancreatitis [143]. While these studies offer critical insights toward the potential therapeutic repurposing of PARP inhibitors in treating non-oncological indications, it is important to learn more about the possible side-effects of long-term treatment and whether PARP inhibition may increase the risk of mutagenesis and carcinogenesis. 

## 9. Perspectives and Conclusions

Individualized treatments to patients with recalcitrant cancers in order to achieve cure are urgently needed. Currently, cancer patients have many therapeutic options for treatment; however, the lack of effective and tumor-selective treatment options still remains a major hurdle in the fight against cancer today. This is due to very limited predictive biomarkers that define individual risks of disease recurrence or sensitivity to treatments. For example, a significant number of cancer patients are over-treated in order to possibly attain an improved overall survival in early cancer stage. This “shotgun” approach, however, could have life-threatening consequences to cancer patients due to the emergence of drug resistance and secondary malignancies. Clearly, there is an urgent need for (1) a clear understanding of normal versus cancer cell biology to gain more informative decisions regarding the most effective therapeutic interventions, and (2) an arsenal of new treatment options based on predictive biomarkers for this disease to eliminate toxic effects to normal cells, drug resistance, and development of secondary cancers.

In less than two decades, studies on the use of PARP inhibitors for the treatment of *gBRCA1/2* mutant cancers and beyond BRCA-deficiency have increased tremendously. Of course, these were all made possible by the collaborative effort of the scientific and medical communities to understand the functional roles of breast cancer associated genes 1 and 2 (BRCA1/2) in normal physiology and breast cancer development. Accordingly, deficiencies in BRCA1 and BRCA2 genes can compromise HR-mediated repair, conferring hypersensitivity to PARP inhibitors by chemical synthetic lethality. The rarity of breast (5–10%) and ovarian (15–20%) cancers with germline *BRCA1/2* loss-of-function mutations, however, currently restricts the therapeutic utility of PARP inhibitor monotherapy. This is further complicated by several reports of potential molecular mechanisms of resistance for PARP inhibitors in BRCA-deficient breast cancers [144]. Interestingly, approximately 24% of high-grade triple negative breast cancers (TNBC) without a *BRCA* mutation showed great response to PARP inhibitor in Phase II clinical trials suggesting that the use of PARP inhibitors can be expanded beyond BRCA deficiency, and this has been reinforced by several studies in other cancers [18,19,20,21]. Indeed, genetic and pharmacological alterations of specific proteins (Table 1) or long non-coding RNAs (lncRNAs, e.g., PCAT-1 [145]) that can induce a state of “BRCAness” or HR-deficiency in BRCA-proficient cancers have the potential to broaden the application of PARP inhibitors beyond the current protocols approved by the FDA and EMA.

In conclusion, a serious concern for the use of PARP inhibitors – as with all chemotherapeutic agents – is the development of acquired drug resistance and de novo malignancies [146] due to limited information and often unclear understanding of the full extent of the agents’ specificity and mechanism of action. A complete and clear knowledge of how different PARPs are activated to perform overlapping and non-overlapping functions or how PAR and NAD^+^ levels are modulated to alter specific biological events toward cellular survival or death are essential to: (1) design novel PARP inhibitors that are more specific and tumor-selective; (2) develop better mechanistic-based strategies using reliable predictive biomarkers for PARP inhibitor monotherapy as well as combination treatments; and (3) identify situations that can re-sensitize recalcitrant tumor cells to PARP activity inhibition or modulation to treat cancer patients more safely and efficiently.

**Table 1:** Representative list of genes/proteins that have been shown to cause “BRCAness” or HR defect in BRCA-proficient tumors when expression is lost or activity is inhibited. These deficiencies could be exploited as predictive biomarkers for precision treatment with PARP inhibitors.

Protein Name	Primary Function/Activity	Association with “BRCAness”	Reference
CDK1	Cell cycle regulation	Loss of expression or activity inhibition compromises phosphorylation of BRCA1 for proper HR function	[30]
CDK12/13	Phosphorylates RNAPII CTD	Loss of expression and activity inhibition suppresses expression of specific HR proteins such as RAD51 and BRCA1	[147]
AXL	A receptor tyrosine kinase associated with metastasis, invasion and migration in many cancers	Loss of expression or activity inhibition decreases expression of specific HR genes and proteins	[148]
Kub5-Hera, RPRD1B, CREPT	Transcription termination factor	Loss of expression compromises HR by decreasing CDK1 expression	[25]
WEE1	Involved in the terminal phosphorylation and inactivation of CDK1-bound cyclin B	Activity inhibition with AZD1775 indirectly inhibits BRCA2	[149]
UCHL3	Deubiquitinase	Activity inhibition with perifosine promotes ubiquitination of RAD51 and blocks the binding of RAD51 with BRCA2	[150]
BET	Transcriptional regulators	Activity inhibition with JQ1 decreases expression of RAD51 and Ku80	[151]
PI3K	Kinase involve in cell growth, proliferation, differentiation, motility, survival and intracellular trafficking	Inhibition of activity impairs BRCA1/2 expression	[152]
Cyclin D1	Regulator of CDKs (cyclin dependent kinases), required for cell cycle G1/S transition	Loss of expression impairs recruitment of RAD51	[153,154]
AURKA	Play important role in mitosis/ regulation of cell cycle progression	Activity inhibition or loss of expression decreases expression of BRCA1 and BRCA2	[155,156]
HKMT	Regulation of histone methylation	Inhibition of activity abolishes retention of BRCA1/BARD1 complexes at sites of DSB	[157,158]
CCDC6	Tumor suppressor	Loss of expression compromises BRCC3 and DNA damage checkpoints in response to DNA damage.	[158,159]
MEK	Kinase that phosphorylates and activates MAPK	Activity inhibition or loss of expression downregulates BRCA2	[160]
HDAC	Removes acetyl groups from an amino acid on a histone	Activity inhibition with SAHA reduces BRCA1 protein levels by targeting the UHRF1/BRCA1 protein complex	[161]
PAK1	Regulates cytoskeleton remodeling, phenotypic signaling and gene expression	Reduced activity and loss of expression downregulates the expression of genes involved in FA/BRCA pathway	[162]
Androgen receptor	DNA-binding transcription factor that regulates gene expression	Activity inhibition or loss of expression suppresses the expression of HR genes, thus creating HR deficiency and BRCAness	[163]
TGFβ	Involved in embryonic development, cell proliferation, motility and apoptosis, extracellular matrix production, and immunomodulation	Overexpression suppresses BRCA1, ATM, and MSH2	[164]
